# Effectiveness of a telehealth-delivered clinician-supported exercise and weight loss program for hip osteoarthritis – protocol for the *Better Hip* randomised controlled trial

**DOI:** 10.1186/s12891-023-07131-0

**Published:** 2024-02-13

**Authors:** Kim L. Bennell, Catherine Keating, Belinda Lawford, Bridget Graham, Michelle Hall, Julie A. Simpson, Fiona McManus, Brinley Hosking, Priya Sumithran, Anthony Harris, Maame Esi Woode, Jill J. Francis, Jennifer Marlow, Sharon Poh, Rana S. Hinman

**Affiliations:** 1 Centre for Health, Exercise and Sports Medicine, Department of Physiotherapy, The University of Melbourne, Vic, Melbourne, Australia; 2 Medibank Private, Vic, Melbourne, Australia; 3 Centre for Epidemiology and Biostatistics, Melbourne School of Population and Global Health, The University of Melbourne, Vic, Melbourne, Australia; 4 Department of Medicine, The University of Melbourne, Vic, Melbourne, Australia; 5 Department of Endocrinology, Austin Health, Vic, Melbourne, Australia; 6 Centre for Health Economics, Monash University, Vic, Melbourne, Australia; 7 School of Health Sciences, The University of Melbourne, Vic, Melbourne, Australia; 8 Centre for Implementation Research, Ottawa Hospital Research Institute, Ottawa, Ontario Canada

**Keywords:** Osteoarthritis, Rehabilitation, Hip, Telehealth, Diet, Physiotherapy, Exercise, Physical activity, Clinical trial, Pain

## Abstract

**Background:**

Hip osteoarthritis (OA) is a leading cause of chronic pain and disability worldwide. Self-management is vital with education, exercise and weight loss core recommended treatments. However, evidence-practice gaps exist, and service models that increase patient accessibility to clinicians who can support lifestyle management are needed. The primary aim of this study is to determine the effectiveness of a telehealth-delivered clinician-supported exercise and weight loss program (*Better Hip*) on the primary outcomes of hip pain on walking and physical function at 6 months, compared with an information-only control for people with hip OA.

**Methods:**

A two-arm, parallel-design, superiority pragmatic randomised controlled trial. 212 members from a health insurance fund aged 45 years and over, with painful hip OA will be recruited. Participants will be randomly allocated to receive: i) *Better Hip*; or ii) web-based information only (control). Participants randomised to the *Better Hip* program will have six videoconferencing physiotherapist consultations for education about OA, prescription of individualised home-based strengthening and physical activity programs, behaviour change support, and facilitation of other self-management strategies. Those with a body mass index > 27 kg/m^2^, aged < 80 years and no specific health conditions, will also be offered six videoconferencing dietitian consultations to undertake a weight loss program. Participants in the control group will be provided with similar educational information about managing hip OA via a custom website. All participants will be reassessed at 6 and 12 months. Primary outcomes are hip pain on walking and physical function. Secondary outcomes include measures of pain; hip function; weight; health-related quality of life; physical activity levels; global change in hip problem; willingness to undergo hip replacement surgery; rates of hip replacement; and use of oral pain medications. A health economic evaluation at 12 months will be conducted and reported separately.

**Discussion:**

Findings will determine whether a telehealth-delivered clinician-supported lifestyle management program including education, exercise/physical activity and, for those with overweight or obesity, weight loss, is more effective than information only in people with hip OA. Results will inform the implementation of such programs to increase access to core recommended treatments.

**Trial registration:**

Australia New Zealand Clinical Trials Registry **(**ACTRN12622000461796).

## Background

Osteoarthritis (OA) is a leading cause of chronic pain and disability worldwide and is strongly linked to many other health conditions such as obesity, diabetes and heart disease [1]. Around 2.1 million Australians have OA, with a 58% increase expected by 2032 due to an ageing population and escalating obesity rates [2]. Osteoarthritis at the hip is common, affecting one in four adults over their lifetime [3]. In 2012, Australian expenditure on OA was $3.75 billion, [2] of which hip replacement for end-stage disease was a major contributor. Of the 38,606 primary hip replacements performed in Australia in 2020, 77% were in people with overweight/obesity [4]. Clinical guidelines emphasise lifestyle management, including education, exercise and weight loss (for those with overweight/obesity) as core OA treatments [5, 6] because they improve pain, function and quality of life [7] and reduce need for hip replacement [8].

Osteoarthritis is predominately managed in the community, yet community care is inadequate, with 57% of people receiving inappropriate care [9]. For many, access to healthcare and support for lifestyle management is challenging, especially in regional and remote regions, where services can be limited or non-existent. Under-utilisation of lifestyle management and over-utilisation of drugs (including opioids) and surgery are unsustainable problems [10, 11]. For example, our analysis of general practitioner (GP) data from 2010 to 2016 (2598 consultations for hip OA [12]) found prescription rates for drugs were much higher than lifestyle management (75 vs 20 per 100 hip OA encounters). Most referrals were to orthopaedic surgeons (65%) with few to physiotherapists (19%) and even fewer to dietitians (0.5%). Meta-analysis [13] shows under-utilisation of lifestyle treatment for OA is a global problem.

There are many barriers to uptake of, and engagement with, exercise and weight loss for people with OA [14, 15] Pain, fatigue and stiffness hinder engagement with exercise. People with OA tend to have a sense that their physical capabilities are limited, and this then becomes self-fulfilling when their physical fitness declines due to decreased activity [16]. A lack of knowledge about the beneficial effects of exercise on OA symptoms and disease progression persists in people with OA, with exercise tending to be viewed as not effective as a treatment, or even harmful to the joints [14, 16]. These inaccurate beliefs hinder people with OA from being physically active. Barriers to engagement with exercise also include a lack of motivation and prioritisation of exercise, feelings of resignation and helplessness about the disease, and a lack of support from health professionals [14, 16]. Lack of motivation and support from health professionals also have been reported as the greatest barriers to weight loss in people with OA [15]. Effective communication and clinician support are vital to correct misperceptions and to improve self-belief and motivation [17], yet clinicians often adopt a biomedical framework that is not patient-centered and does not facilitate self-management [15]. We identified many barriers to implementing high value OA care amongst 1886 clinicians (GPs, nurses, physiotherapists) [18], including limited skills and confidence in exercise, nutrition, and behavioural counselling. Further, people with hip OA, especially in regional and rural areas, have difficulty accessing physiotherapists, (e.g. 101 physiotherapists/100,000 people in Australian cities, compared to just 38 in remote areas) and dietitians (e.g. only 5071 accredited practicing dietitians in Australia, mostly urban). Even in metropolitan areas, travelling to consult a clinician can be difficult for people with OA due to pain and restricted mobility. Accordingly, there are calls for increased implementation of evidence-based telehealth OA services to improve access to care and reduce inequity [19].

A comprehensive multi-disciplinary program that increases access to best-practice exercise and nutrition care is required for people with hip OA. Telehealth offers an acceptable, equitable and sustainable delivery mode to achieve this aim. *Better Hip* was adapted from a program we developed for knee OA (Better Knee, Better Me) that was shown to be effective in improving pain and function in a randomised controlled trial (RCT) [20, 21] and which has now been rolled out by a major Australian health insurer (Medibank) to their members. With end-users we adapted *Better Knee, Better Me* for hip OA by asking four physiotherapists for their input on modifications to the physiotherapy protocol and sought consumer and Medibank feedback on changes to the participant resources to make them targeted to hip OA and weight loss in this population. We then piloted the *Better Hip* program in 18 people. Retention was excellent with 89% completing 6-month follow-up. All attended all 6 dietitian consults and 89% attended ≥5 of the six physiotherapy consults. Most (88%) were “extremely satisfied” with the program [22]. Participants in the pilot were interviewed, and their experiences were used to refine the final *Better Hip* program. *Better Hip* incorporates education, exercise/physical activity, self-management support and, for those who need it, dietary intervention for weight loss (a ketogenic very low energy diet (VLED) demonstrated to be effective for achieving rapid and substantial weight loss) [23]. The program includes videoconference consultations with physiotherapists and dietitians, behaviour change support, and education/resources to facilitate sustained exercise and nutrition lifestyle behaviour change by patients.

Thus, this study aims to determine the effectiveness of the *Better Hip* telehealth-delivered clinician-supported program on the primary outcomes of change in hip pain on walking and change in physical function at 6 months, compared with an information-only control, for people with hip OA. We also aim to determine the effectiveness of the *Better Hip* program on primary outcomes at 12 months and secondary outcomes at 6- and 12-months as well as conduct a cost effectiveness analysis of the program at 12 months.

## Methods

### Study design

The *Better Hip* trial is a two-group, parallel-design, superiority pragmatic RCT conducted across Australia, with nested qualitative studies and health economic evaluation. The trial is designed according to SPIRIT (Standard Protocol Items: Recommendations for Interventional Trials) guidelines [24] and principles of Good Clinical Practice. It has been prospectively registered (ACTRN12622000461796) and will be reported according to the CONSORT statement and relevant extensions [25]. A study Data Safety and Monitoring Committee has been established consisting of a doctor with expertise in weight loss, a biostatistician and a physiotherapist in clinical practice, none of whom are involved in the study, have any conflicts of interest, or will benefit from the results. The committee will receive a study report every 3 months but will not meet unless warranted. There is no planned interim analysis or stopping guidelines. We will describe any protocol amendments in our internal trial protocol document, notify the institutional ethics committee and if appropriate update the trial registry.

### Participants

We will recruit community participants from members of one of Australia’s largest private health insurers, Medibank Private Limited (Medibank) (approximately 3.7 million members). A total of 212 participants across Australia with chronic hip pain consistent with a clinical diagnosis of hip OA [6] will be recruited via marketing campaigns run by Medibank, leveraging channels such as its website, email newsletters, health promotion emails and social media.

Inclusion criteria are as follows:

i) National Institute for Health and Care Excellence [6] clinical criteria for OA:
  age ≥ 45 years;
  activity-related hip joint pain; and
  no morning hip stiffness, or morning hip stiffness ≤30 mins;

ii) report hip pain on most days for ≥3 months;
iii) report average hip pain during walking in the past week ≥4 on an 11-point numerical rating scale (NRS; 0 = no pain, 10 = worst pain possible);
iv) access to a computer/laptop/tablet with internet connection and a webcam for videoconferencing consultations;
v) own a smartphone (for pairing with activity tracker);
vi) willing and able to participate in video consultations for physiotherapy and dietitian appointments;
vii) member of Medibank with a level of cover that includes total joint replacement surgery; and
viii) able to give informed consent and to participate in the interventions and assessment procedures.

Exclusion criteria are as follows:

i) unable to speak or read English;
ii) on waiting list for/planning knee/hip surgery in next 6 months;
iii) previous joint replacement on affected hip;
iv) recent hip surgery (past 6 months);
v) doing regular leg strengthening exercise (at least once per week) each week for the past 6 weeks;
vi) weight loss of > 2 kg over the past 3 months;
vii) currently participating in a weight loss intervention;
viii) planned bariatric surgery in next 6 months;
ix) pregnancy or planned pregnancy;
x) self-reported inflammatory arthritis (e.g. rheumatoid arthritis);
xi) neurological condition affecting lower limbs;
xii) unstable/uncontrolled cardiovascular condition;
xiii) fall/s history (past 12 months) without GP clearance to participate;
xiv) house-bound due to immobility without GP clearance to participate; and/or
xv) answering ‘yes’ to any of the Exercise and Sports Science Australia stage 1 pre-exercise screening questions [26] without GP clearance to participate.

A clearance letter to participate in the study signed by a doctor is required from anyone who i) reports a fall (past 12 months); ii) is house-bound due to immobility; and/or iii) answers ‘yes’ to any of the Exercise and Sports Science Australia stage 1 pre-exercise screening questions.

### Procedures overview

**Fig. 1:**
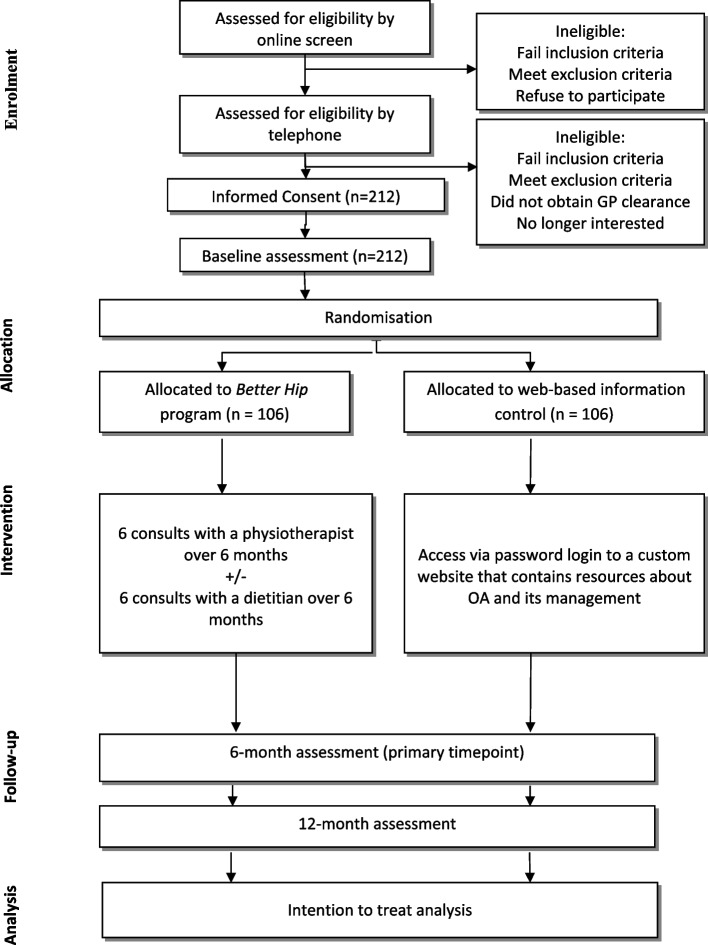
Participant flow through the randomised controlled trial

The trial phases are summarized in Fig. 1. Medibank will provide members with details to access an online questionnaire that will help determine preliminary eligibility. People deemed potentially eligible will then undergo telephone screening by the research staff to further explain the study, confirm eligibility, and obtain verbal consent. Those with body mass index (BMI) > 27 kg/m^2^ will be asked to confirm their willingness to participate in the dietary intervention in the event that they are allocated to that group, and ascertain if they have conditions that preclude them from being on the VLED. A pre-exercise screening survey (Exercise & Sports Science Australia Adult Pre-Exercise Screening System) [26] will be included in the phone screen questionnaire to identify individuals who may be at an increased risk of falling or an adverse event related to exercising and therefore may require further assessment and GP clearance. Potential participants will be sent the Plain Language Statement (PLS) and Consent Form by email. If they have any questions or concerns regarding the contents of the PLS and/or Consent Form they will be encouraged to phone the researchers. Participants will provide consent online using REDCap prior to completing the baseline questionnaire or they will sign a paper-based consent form and return it via a reply-paid envelope in the post or by scanning and emailing the document to the Trial Coordinator. For participants with bilateral hip pain, the most symptomatic eligible hip will be deemed the study hip with respect to the exercise intervention and outcome measurement. Throughout the trial, participants will not be restricted from using co-interventions and use of these will be measured as described below.

### Randomisation, blinding and allocation concealment

Eligible participants will be randomised to receive either i) *Better Hip* program, or ii) web-based information (control). The randomisation schedule was computer generated by an independent biostatistician, using permuted random block sizes, stratified by participant BMI (> 27 kg/m^2^ and ≤ 27 kg/m^2^). The randomisation schedule is stored on a password-protected website (REDCap) at the University of Melbourne and will be maintained by a researcher not involved in either recruitment of participants or administration of primary/secondary outcome measures. Group allocation will be revealed by this same researcher after baseline assessment has been completed. Participants allocated to the *Better Hip* group will be randomly allocated to a physiotherapist and those who are eligible and choose to undergo dietary intervention will also be randomly allocated to a dietitian.

As this is a pragmatic trial, participants will not be blinded to group allocation. As participants are not blinded and the primary and secondary outcomes are participant-reported, by default the assessors of these outcomes are not blinded. Research staff administering and entering any participant-reported data will be blinded. The statistical analysis plan will be written and published while the biostatisticians are blinded. Main statistical analyses will be performed blinded to intervention group name.

### Clinicians and training

Six physiotherapists and four dietitians were recruited and provided with standardised training, detailed trial manuals, videos and practice sessions aligned with best practices and recommendations by the National Institutes of Health Behaviour Change Consortium [27]. All clinicians will be involved in regular meetings with research staff throughout the trial to prevent skill drift.

Physiotherapist training included:

- Custom-designed self-directed e-learning modules on best-practice OA management, telehealth delivery and trial procedures, including the physiotherapy treatment protocol (estimated time commitment of 5 hours);
- Live online tutorial in how to use Coviu online telehealth platform (estimated time commitment of 1 hour);
- Participation in a mock initial video consultation (competency check) with research staff (estimated time commitment of 1 hour);
- Undertaking four video consultations with two pilot patients (an initial and a follow-up consultation for each patient) to practice video consultation skills (estimated time commitment of 2.5 hours);
- Participation in a teleconference with research staff (estimated time commitment 30 minutes) to answer any questions and clarify any procedures.

Dietitian training included:

- Custom-designed self-directed e-learning modules on best-practice OA management, telehealth delivery and trial procedures, including the dietetics treatment protocol (estimated time commitment of 5 hours);
- 2.5 day face-to-face motivational interviewing course (run by Health & Wellbeing Training Consultants https://www.thinkhealthwellbeing.com.au/our-services/motivational-interviewing/) for effective patient education and facilitation of behaviour change;
- Live online tutorial in how to use the online telehealth platform (estimated time commitment of 1 hour);
- Participation in a mock initial video consultation (competency check) with research staff (estimated time commitment of 1 hour);
- Undertaking four video consultations with two pilot patients (an initial and a follow-up consultation for each patient) to practice video consultation skills (estimated time commitment of 2.5 hours);
- Participation in a teleconference with research staff (estimated time commitment 30 minutes) to answer any questions and clarify any procedures;
- VLED/ketogenic diet training provided in a 1-hour webinar following pre-reading of an article on the specifics of the diet.

### Interventions

- *Better Hip* program

The *Better Hip* program includes components based on best-practice for managing hip OA [5] and obesity [28]. The personalised program is underpinned by the Chronic Care model [29], recommendations for design and evaluation of self-management programs [30] and behaviour change theory [31] and was adapted from our *Better Knee, Better Me* program for knee OA [21]. The program aims to act through: i) improving knowledge and motivation regarding effective self-management; ii) increasing self-efficacy regarding OA self-management and diet control; iii) positive treatment beliefs about exercise and weight loss; and iv) positive outcome expectations from recommended treatment.

The *Better Hip* program embeds key behaviour change techniques as per Michie et al. [32] that are known to be effective for exercise and eating behaviours. These include goal setting, behavioural monitoring, feedback, positive reinforcement for progress, instruction, social support, problem solving, and action planning (Table 1).

**Table 1:**
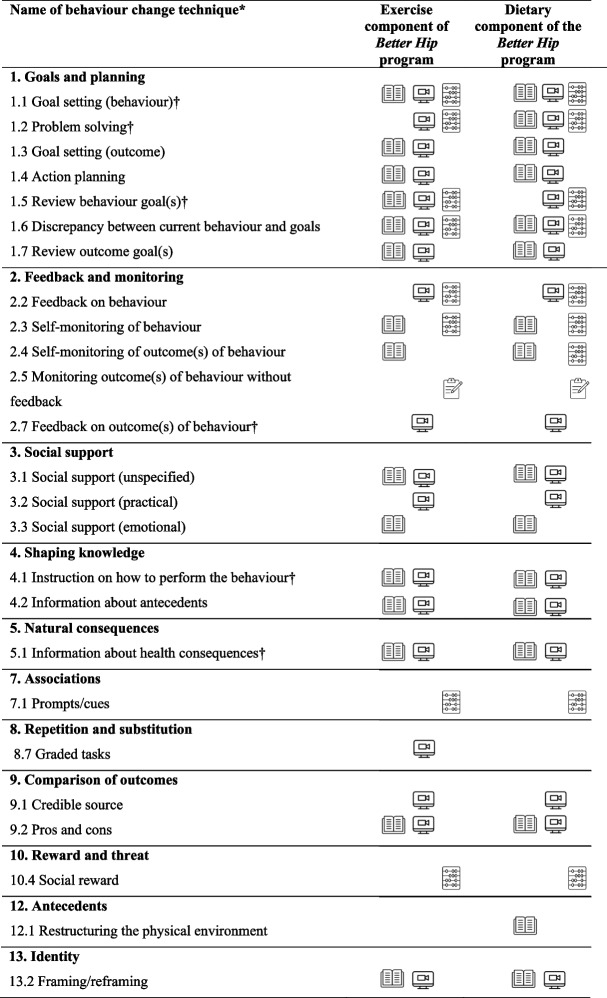
Main behaviour change techniques incorporated into the program**

* Based on the Behaviour Change Technique Taxonomy v1 [32]

† Core behaviour change techniques, delivery audited by clinician self-report

Included/incorporated into printed participant resource booklets

Provided by clinician during video consultations

Incorporated into trial procedures (e.g. written communication to participant’s GP about their progress)

Physical resources provided to participants (e.g. activity tracker, portion plates, My Exercise Messages app)

** Adapted from: Bennell KL, Lawford BJ, Keating C, et al. Comparing Video-Based, Telehealth-Delivered Exercise and Weight Loss Programs With Online Education on Outcomes of Knee Osteoarthritis : A Randomized Trial. Ann Intern Med. Feb 2022;175(2):198-209

*Better Hip* will be delivered by physiotherapists and dietitians using Coviu, a specialised telehealth video conferencing platform (www.coviu.com/en-au), accompanied by a suite of educational resources in hard copy. With the participant’s consent, physiotherapists and dietitians will provide written communication to the participant’s GP about their progress and outcomes.

#### Exercise/physical activity intervention

All participants will have six individual consultations with a physiotherapist. Consultations will be recommended to occur in weeks 1, 3, 7, 11, 16, and 21, but the exact timing will be discussed between each participant and their physiotherapist. The consultations will cover education about OA and discussion of non-drug treatment options, prescription of an individualised home-based strengthening program (to be undertaken three times per week) and physical activity plan, behaviour change support, and facilitation of other self-management strategies. Participants will also receive exercise resistance bands, an adjustable cuff weight, exercise/activity log sheets and a wearable activity tracker to allow them to track and monitor their physical activity.

Prior to the initial consultation, participants will complete a pre-consultation survey asking them about their main problems and goals, and a brief history about their hip and other health problems. This will be provided to the clinicians prior to their first consultation with the participant, with the aim of making the first consultation more informed and efficient.

The initial physiotherapy consultation will be approximately 45 minutes in duration, and follow-up consultations will be approximately 30 minutes. During the initial consultations, a shared decision-making approach will be used to formulate appropriate goals and a tailored management plan. A custom-designed support “option grid” will be used to assist with shared decision-making regarding hip OA management options, and information sharing between the participant and physiotherapist. Education will be provided from a biopsychosocial perspective and will contain positive messaging to promote hope and optimism for the future, positive expectation of outcomes from active self-management strategies, and confidence in moving with and managing pain. Education will include discussion of a range of available interventions. Accurate information about the likelihood of future need for surgery will be provided and reasonable expectations about the role of hip joint replacement surgery in hip OA management will be established.

Participants’ management plans will include the following components:

- A structured, personalised and progressive exercise program aimed specifically at increasing muscle strength;
- A tailored physical activity plan to increase incidental and general physical activity and reduce sedentary behaviour, including a daily step goal;
- Education and advice about other practical self-management strategies (e.g. activity modification, activity pacing, pain coping strategies, sleep advice);
- Customised education materials delivered in hard copy.

Based on their assessment of, and in discussion with, the participant, physiotherapists will prescribe 5 to 7 strengthening exercises primarily targeting the study leg from a pre-determined list and to be performed at home at least three times/week (see Appendix 1). During the consultations, the physiotherapists can provide real-time demonstration of exercises to participants via a bespoke website containing a video library of exercises contained within the “Exercise Booklet” and using the share-screen feature of Coviu. The target intensity for the strengthening exercises is 5 to 7 out of 10 (hard to very hard) on the modified Borg Rating of Perceived Exertion CR-10 scale [33] for strength training. Participants will be instructed that each exercise should be performed slowly and in a controlled manner. Progression will be guided in accordance with American College of Sports Medicine guidelines [34] by adjustments to repetitions, direction, and speed of movements; increasing resistance; and/or changing stance surface.

Regular follow-up consultations between the participant and the physiotherapist to review progress, goals and the management plan and make modifications as required to ensure effective adoption of positive lifestyle changes and self-management strategies. The physiotherapist will also provide behaviour change support and assistance to overcoming obstacles to the participant enacting the agreed management plan.

### Diet intervention

If participants have a BMI > 27 kg/m^2^, are aged < 80 years and do not have specific conditions which are likely to require medical supervision to participate in a ketogenic VLED (type 1 diabetes, type 2 diabetes requiring medication apart from metformin, using warfarin, stroke or cardiac event in the previous 6 months, unstable arrhythmia, constipation requiring medical intervention in the past 12 months, restriction of fluid intake or renal problems with estimated glomerular filtration rate < 30 mL/min/1.73m^2^), they can also opt to have six individual videoconferencing consultations with a dietitian to undertake a dietary weight loss intervention [20, 21]. Participants must also be willing to undertake the VLED using the meal replacement products to be eligible for the dietitian consultations.

The initial dietitian consultation will be approximately 45 minutes in duration with follow-up consultations approximately 30 minutes. It is recommended that the six consultations occur in weeks 2, 4, 6, 9 to 12, 14 to 17 and 19 to 23 but the exact timing will be agreed between each participant and their dietitian. During the consultations, appropriate weight loss goals will be agreed and a tailored management plan for losing weight developed. Conversations based on motivational interviewing principles and techniques will be used to develop readiness to change (motivation) and confidence to self-manage. Ongoing education, information and advice are key components of the intervention to optimally support weight loss. The dietitian will use follow-up consultations to review progress, goals and weight loss progress and together with the participant make modifications as required. The dietitian will assist in providing behaviour change support and assistance to overcome obstacles to the participant enacting the agreed weight loss plans. Core behaviour change techniques employed by the dietitian are specified in Table 1.

The diet intervention comprises two phases:

1. Intensive weight loss through a ketogenic VLED [35] for up to 16 weeks and aiming for ≥10% loss of body weight, as this is associated with clinically important improvements in knee OA pain and function [36, 37]. The ketogenic VLED involves replacing two meals, generally breakfast and lunch, with formulated meal replacement products (Optifast® meal replacements, or if unavailable or the participant is vegetarian, Optislim®) provided at no cost to the participant. These products provide most of the vitamins, minerals, and metals required for optimal nutrition, and come as soups, shakes and bars in various flavours. On the diet, one prepared meal (generally dinner) comprises protein (e.g. white or red meat, fish or seafood, eggs, or tofu) and non-starchy vegetables/salad. A small amount (i.e. 1 tablespoon) of fat/oil is also recommended for this meal to stimulate gallbladder contraction (if the gallbladder is in situ). In total, the diet contains approximately 800 cal (3280 kJ) per day and < 60 g of carbohydrates.
2. Transition from ketogenic VLED onto a longer-term eating plan for weight maintenance. After 16 weeks (or earlier if the participant has reached their weight loss target), participants will be guided to progress to one meal replacement per day for a further 4 weeks (supplied at no cost to the participant) and to reintroduce foods containing carbohydrates, to aid the gradual transition to a weight maintenance phase. Participants will be advised to follow a healthy eating diet consistent with the principles of the Commonwealth Scientific and Industrial Research Organisation total wellbeing diet [38] (i.e. high protein, low glycaemic index carbohydrate, low fat). Participants are encouraged to continue to weigh themselves regularly (e.g. at least once per week) and to restart the ketogenic VLED taking meal replacements for 1 to 2 weeks if they regain 2 kgs or more. The dietitian will refer the participant to see their general practitioner for a health check if they lose > 20% of their body weight within 6 months, or for monitoring of known health problems if deemed necessary (e.g. hypertension).

#### Enabling and behaviour change strategies/resources (see Tables 1 and 2)

Participants will be provided with resources to facilitate the management plans the physiotherapists will deliver during the sessions. These resources include: a welcome letter describing their involvement in the study; hard copy information booklets; four coloured therabands (red, green, blue, and black) and an adjustable cuff weight (0.5 to 5 kg) for performing strengthening exercises; and a wearable activity tracker to monitor daily step count.

**Table 2:** Summary of resources provided to participants in the *Better Hip* group^a^

Resource	Description	*Better Hip* program (eligible for weight loss intervention)	*Better Hip* program (not eligible for weight loss intervention)
Consultations with a physiotherapist	6 videoconferencing consultations over 6 months. Prescribes structured exercise and physical activity plan and behaviour change support	✓	✓
Consultations with a dietitian	6 videoconferencing consultations over 6 months. Supports participant to undertake ketogenic VLED, including behaviour change support	✓	
Exercise bands	4 exercise resistance bands (red, green, blue, black) for strengthening exercises	✓	✓
Exercise weights	Adjustable ankle cuff weight (0.5 kg – 5 kg) for strengthening exercises	✓	✓
Fitbit® activity tracker	Activity tracker used to track participants’ steps and physical activity	✓	✓
Access to My Exercise Messages mobile app	Mobile app which tracks weekly exercise sessions and provides personalised messages to help overcome obstacles to exercise	✓	✓
Digital weight scales	Provided to those who do not already have access to scales	✓	
VLED plastic portion plate	Assists with portion sizes (for use during the VLED phase of the dietary intervention)	✓	
Weight maintenance plastic portion plate	Assists with portion sizes (for use during the weight maintenance phase of the dietary intervention)	✓	
Optifast® meal replacements	Up to 6 months supply of meal replacements for the ketogenic VLED	✓	
Educational video about the VLED	Short video about the ketogenic VLED featuring endocrinologists and dietitian experts, and a person with OA	✓	
Booklets			
Preparing for your consultations	Details about consultations, instruction on how to use Coviu videoconferencing	✓	✓
Osteoarthritis information	Information about osteoarthritis, typical management options, weight loss, pain coping skills and sleep	✓	✓
Exercise booklet	Strengthening exercise instructions and photographs	✓	✓
Hip care plan and log book	Templates to record details of management plans and complete exercises	✓	✓
Weight management ‘how to’ guide	Describes the ketogenic VLED and provides information about healthy food choices and portion sizes	✓	
Weight management behavioural support activities	Workbook that contains information and templates to track weight, a food diary, tips to find a support person, identify food triggers, plans for ‘at risk’ scenarios, overcoming barriers, changing thought patterns, and monitoring hunger levels	✓	
Recipe book	Suitable recipes for ketogenic VLED	✓	
Food list pocket guide	Low carbohydrate ingredients to consume when on the ketogenic VLED	✓	

*VLED* Very low energy diet, *OA* Osteoarthritis.

^a^Adapted from: Bennell KL, Keating C, Lawford BJ, et al. Better Knee, Better Me™: effectiveness of two scalable health care interventions supporting self-management for knee osteoarthritis–protocol for a randomized controlled trial. BMC musculoskeletal disorders. 2020;21(1):1–19

Physiotherapists will advise the participants to download the ‘My Exercise Messages’ app [39] which we developed and which is freely available from the Apple Store or Google Play. ‘My Exercise Messages’ was created to help support people with OA stick to their weekly exercise goals, including exercise prescribed by health professionals. The app works by tracking weekly exercise sessions and providing personalised messages to help overcome obstacles to exercise, and was based on a Short Message Service (SMS) program which was proven to enhance exercise adherence in an OA population [40]. Physiotherapists will advise the participants to use the app to track performance of their strengthening exercise program.

Additional resources for those undertaking weight management include:

- A plastic “portion plate” to help manage meal portion sizes and stick to the ketogenic VLED when preparing meals;
- Another plastic “portion plate” to help manage meal portion sizes when transitioning to the healthy eating diet for weight maintenance;
- A set of digital weight scales for those who do not already have access to scales;
- Weight management ‘how to’ guide describing the ketogenic VLED, healthy food choices and portion sizes;
- Ketogenic recipe book;
- Food list pocket guide;
- Workbook with information and templates to track weight, a food diary, tips to find a support person, identifying food triggers, planning for “at risk” situations, overcoming barriers, changing thought patterns, and monitoring hunger levels.

Resources and strategies that the dietitian will use to provide behaviour change support include: setting realistic goals; keeping a food diary; monitoring weight regularly (at least weekly); finding a support person to help; learning about healthy food choices and portion size; identifying food triggers; planning for ‘at risk’ situations; working out barriers and finding ways to overcome them; changing any negative thought patterns; engaging in relaxation, mindfulness and distraction techniques; monitoring hunger levels before, during and after meals to help identify physical and psychological hungers and strategies to overcome psychological hunger.

### Web-based information (control)

This group will receive online information about hip OA and its management via a custom website accessible during their involvement in the study via password login. The Trial Coordinator will call participants allocated to the control group upon study enrolment to explain group allocation and how to access the website, after which the website URL and login details will be emailed to the participant. The website will include educational information about OA, recommended treatment options, exercise and physical activity, weight loss, managing pain, and optimising sleep, as well as links to external websites for further help and support (e.g. MyJointPain, painHEALTH, Musculoskeletal Australia).

### Outcome measures

Participant-reported outcomes will be collected online via REDCap data capture platform at baseline, 6 months and 12 months. All participants who complete the 12-month re-assessment will be given a $50 gift voucher as compensation for the considerable time they have invested in the trial. An online survey for follow-up of primary and secondary outcomes will also be conducted at 2 years post randomisation. These longer-term results will be reported separately to the main trial results and will occur after analysis of the main trial data.

Primary outcomes are reliable and valid measures recommended for use in clinical trials of hip OA [41]. These will be measured at baseline, 6 and 12 months, and include i) change in average severity of hip pain on walking in the past week measured on an 11-point NRS, where 0 = “no pain” and 10 = “worst pain possible”; ii) change in physical function subscale of the Western Ontario and McMaster Universities Osteoarthritis Index (WOMAC) [42] with scores from 0 to 68, where higher scores indicate greater dysfunction. Conclusions about effectiveness will be based on the 6 month time point change in the primary outcomes.

The secondary outcomes will be measured at baseline, 6 and 12 months unless otherwise indicated and include:

i) Change in Hip dysfunction and Osteoarthritis Outcome Score (HOOS) [43] subscale of pain with normalised scores ranging from 0 to 100, with 100 indicating no symptoms;
ii) Change in HOOS [43] subscale of hip-related quality of life, with normalised scores ranging from 0 to 100, with 100 indicating better quality of life;
iii) Change in HOOS [43] subscale of function, sports and recreational activities with normalised scores ranging from 0 to 100, with 100 indicating better function;
iv) Change in self-reported body weight measured in kilograms;
v) Change in health-related quality of life using the Assessment of Quality of Life instrument (AQoL-8D) [44], a 35-item instrument with scores ranging from − 0.04 to 1.0, with higher scores indicating better quality of life;
vi) Change in physical activity levels evaluated using the Physical Activity Scale for the Elderly (PASE) [45] with scores from 0 to 400, where higher scores indicate greater levels of physical activity;
vii) Perceived global rating of overall change in hip condition scored on a 7-point Likert scale from ‘much worse’ to ‘much better’ [46] at 6 and 12 months. Participants who indicate that they are “moderately better” or “much better” will be categorised as ‘improved’;
viii) Self-reported hip replacement procedures, where participants will be asked if they have had a hip joint replacement since their study enrolment on the study hip at 6 and 12 months;
ix) Willingness to undergo hip replacement surgery self-rated using a 5-point Likert scale with anchors “definitely not willing” and “definitely willing”. Participants indicating ‘probably willing’ or ‘definitely willing’ will be classified as ‘willing’;
x) Use of oral pain medications self-reported and defined as one or more of analgesics (paracetamol combinations) and/or oral non-steroidal anti-inflammatory drugs and/or oral glucocorticoids and/or oral opioids taken at least once a week in the prior month for hip pain.

### Other measures

Other measures to be collected at baseline, 6 and 12 months include:

i) Hip Osteoarthritis Knowledge Scale [47], scored from 11 questions on a 5-point Likert scale with scores ranging from 11 to 55 where higher scores indicate greater knowledge about OA;
ii) Attitudes toward self-management using the Patient Activation Measure (PAM-13) [48], scored from 13 statements rated on a 4-point Likert with higher scores indicating greater patient activation;
iii) Brief Fear of Movement Scale for Osteoarthritis [49], scored from 6 statements on a 4-point Likert scale with scores ranging from 6 (minimal fear) to 24 (maximal fear);
iv) Arthritis self-efficacy scale (pain subscale) [50], scored from 5 items on a 10-point NRS with the total presented as an average of the 5 items where higher scores indicate higher self-efficacy.

### Treatment adherence

A number of adherence measures will be collected from participants allocated to the *Better Hip* program. These include:

i) Number of consultations with the physiotherapist, taken from consultation notes (0 to 6), reported as the mean (standard deviation) as well as the number and proportion of participants deemed to be adherent (≥4 consultations);
ii) Duration of physiotherapist consultations (minutes);
iii) Number of eligible participants (BMI > 27 kg/m^2^ and no exclusions for ketogenic VLED) choosing to undergo dietary intervention;
iv) Number of consultations with the dietitian for those undergoing dietary intervention, taken from consultation notes (0 to 6), reported as the mean (standard deviation) as well as number and proportion of participants deemed to be adherent (≥4 consultations);
v) Duration of dietitian consultations (minutes);
vi) Adherence to the strengthening exercise program self-rated at 6 and 12 months on an 11-point NRS in response to the question: “Over the past 6 months, how much did you adhere to the strengthening exercise program provided to you by the physiotherapist?” with responses from 0=“not at all” to 10=“completely as instructed”;
vii) Number of days strengthening exercises were performed in the previous fortnight self-reported at 6 and 12 months;
viii) Perceived intensity of the strengthening exercise program self-rated at 6 months using the modified Borg Rating of Perceived Exertion CR-10 scale [33]. Participants will be asked “When performing your strengthening exercises over the past six months, how hard do you feel you have been working on average?” Scores range from 0 to 10 with higher scores indicating higher perceived intensity of exercise;
ix) Adherence to a physical activity plan self-rated at 6 and 12 months on an 11-point NRS in response to the question: “Over the past 6 months, how much did you adhere to the physical activity plan provided to you by the physiotherapist?” with responses from 0=“not at all” to 10=“completely as instructed”.
x) Adherence to a weight management plan (for those undergoing dietary intervention) self-rated at 6 and 12 months on an 11-point NRS in response to the question: “Over the past 6 months, how much did you adhere to the weight management plan?” with responses from 0=“not at all” to 10=“completely as instructed”.

### Other process measures

A number of other self-reported process measures will be collected at 6 months from the *Better Hip* group only (unless otherwise stated) including: Number of times website accessed (control group only); usefulness of physiotherapist consultations, dietitian consultations, OA educational resources, ‘My Exercise Messages’ app, activity tracker, weight loss program, weight loss/management resources, strengthening exercise program, physical activity plan, exercise/physical activity resources, each scored separately on an 11 point NRS with 0=“not at all useful” to 10 = “extremely useful”; use of ‘My Exercise Messages’ app in response to question “Did you use the ‘My Exercise Messages’ app to help you stick to your strengthening exercise program?” (Yes/No); satisfaction with the *Better Hip* program scored on a 5-point Likert scale with response options from “extremely unsatisfied” to “extremely satisfied” (those scoring “moderately” or “extremely” classified as “satisfied”) and; likelihood of recommending the *Better Hip* program to someone else with the same condition scored on a 5-point Likert scale with response options from “extremely unlikely” to “extremely likely” (those scoring “moderately” or “extremely” classified as “likely”). The control group will also be asked to self-report at 6 and 12 months whether they have undertaken any regular exercise for their hip (defined as 2 or more times per week for at least 1 month) and the type of such exercise.

### Descriptive measures

Baseline self-reported descriptive measures include age, height, BMI, gender, country of birth, ethnicity, geographical location, education level, current employment status, occupation, weekly earnings before tax, symptom duration, time since first visit to doctor for hip pain, comorbidities assessed using the Self-Administered Comorbidity Questionnaire [51], history of hip surgery, and treatment expectation assessed on a 5-point ordinal scale.

### Treatment fidelity

Consultations with physiotherapists and dietitians in the *Better Hip* group will be audio-recorded and files will be stored on a secure password-protected cloud-based system. Bespoke semi-structured consultations notes will be completed online on REDCap™ by physiotherapists and dietitians for each consultation. Notes will be scrutinised by research staff for clinician adherence to protocols.

Fidelity, as determined from the clinician consultation notes, will be reported as the number and proportion of participants where the physiotherapist: i) prescribed a strengthening exercise program; and ii) prescribed a physical activity plan, and where the dietitian: i) discussed the use of meal replacements; ii) discussed ketogenic principles for preparing the third meal during the ketogenic VLED phase; iii) discussed transition to healthy eating; and vi) discussed healthy eating principles. The number and proportion of participants where the clinicians delivered the core behaviour change techniques will be reported for: goal setting; problem solving; review behaviour goals; feedback on behaviour; feedback on outcomes of behaviour; instruction on how to perform the behaviour; and information about health consequences.

### Adverse events

Related adverse events are defined as “any problem experienced in the study hip or elsewhere in the body deemed to be a result of participating in the trial and at least one of i) caused negative/adverse symptoms/effects for two days or more, and/or ii) resulted in the participant seeking treatment from a health professional”. Adverse events will be ascertained by survey questions to participants at 6 and 12 months.

A serious adverse event is defined as any untoward medical occurrence that; i) results in death; ii) is life-threatening; iii) requires hospitalisation or prolongation of existing inpatient hospitalisation; iv) results in persistent or significant disability or incapacity; v) is a congenital anomaly or birth defect, or; vi) any other important medical condition which, although not included in the above, may require medical or surgical intervention to prevent one of the outcomes listed. Due to the low-risk nature of the interventions in this trial, related serious adverse events are extremely unlikely. Participants, dietitians and physiotherapists will be advised to report any serious adverse events to the Trial Coordinator as soon as they can by telephone or email, which will be documented and reported to the Sponsor (University of Melbourne) within 24 hours of the research staff becoming aware of the event.

Any adverse events reported by telephone/email or in questionnaires will be reported to the Internal Trial Monitoring Committee, including the Chief Investigator who will be responsible for deciding what action, if any, is needed on a case-by-case basis. All recorded adverse events will also be reported as blinded data to the Data Safety and Monitoring Committee for the study. The Internal Trial Monitoring Committee and the Data Safety and Monitoring Committee will collectively determine whether reported adverse events are likely to be related to the intervention.

We will report the number and proportion of participants who: withdraw from the study due to a related adverse event; experience one or more serious related adverse events and their types; and experience one or more non-serious related adverse events and their types.

### Health economic measures and evaluation

We will conduct economic evaluations (led by AH) with results reported separately to the main trial outcomes. These will use data from Medicare/Pharmaceutical Benefits Scheme and Medibank and will assess and compare cost-effectiveness of *Better Hip* including: i) cost per extra person with a clinically significant improvement in pain and function; and ii) per quality-adjusted life years (QALY) gained for *Better Hip* group compared to control at 12 months.

Health care expenditure for each participant will be extracted from Medicare Benefit Schedule (MBS), Pharmaceutical Benefit Scheme (PBS) and Medibank data for 12 months prior to baseline and for 24 months after enrolment. The MBS collects information on medical visits and procedures, and the associated costs. The PBS collects information on prescription medicines filled at pharmacies, and Medibank collects information on member hospital, medical and ancillary services for claims made against their Medibank membership. We will seek participants’ consent to access their Medibank data and their MBS and PBS data from the Australian Government Department of Human Services. Public hospital admission/attendance will be self-reported at baseline, 6 and 12 months via a bespoke questionnaire asking number, reason for and length of public hospital admissions in the last 6 months. Participants will also be asked if they attended an emergency department and/or any outpatient appointments at a public hospital.

For those in employment, work productivity will be assessed using the World Health Organisation Health and Work Performance Questionnaire (clinical trials version) [52].

QALYs will be calculated based on utility scores using the AQoL-8D at baseline and 12 months. Difference in health care usage and productivity lost between baseline and 12 months will be compared, as will the association between utility gains (AQoL-8D) and productivity.

### Trial sample size

We aim to detect the minimal clinically important difference (MCID) over 6 months on the two primary outcomes of i) change in hip pain during walking (NRS) and ii) change in physical function (WOMAC). The MCID in OA trials is a 1.8-unit pain change [53] and 6-unit function change [42]. The sample size calculation accounts for potential clustering by physiotherapists in the *Better Hip* group. Based on our research [54], we assume a conservative between-participant standard deviation of 2 pain units and 11 function units, correlations between baseline and 6-month scores of 0.25 for pain and 0.4 for function, an intra-cluster correlation of 0.05 and 5 physiotherapists treating participants. With these parameters, we need 90 per group to achieve 80% power to detect the MCID in function at a 0.025 significance level (due to two primary outcomes). This gives > 99% power to detect the MCID in pain. Allowing for 15% attrition, we will recruit 106 people per group (in total *n* = 212).

### Data analysis plan

We will use intention-to-treat analyses. For the two primary outcomes and other secondary continuous outcomes, mean differences in change over time between groups will be estimated via separate linear mixed-effects models, with random effects for participants and physiotherapist. Models will be adjusted for baseline outcomes and BMI (> 27 kg/m^2^ and ≤ 27 kg/m^2^). Terms for time and treatment will be included, and their interaction, and results reported as mean difference in outcomes at 6 months (primary timepoint) and at 12 months between the *Better Hip* group and control group. These models provide valid inference in the presence of missing data if the data are missing at random. An analysis will be conducted using the delta-adjustment method under the pattern-mixture modelling framework in the context of multiple imputation to assess sensitivity to missingness not at random. To aid clinical interpretation, the primary outcomes will also each be dichotomised into those who do and do not achieve the MCID in improvement in pain (1.8 NRS units) and function (6 WOMAC units). Counts and percentages of participants achieving the MCID in improvement in pain and function will be reported in each treatment group at 6 and 12 months. For binary outcomes (clinically-relevant improvement, global change, participants using any oral pain medication at least once per week, participants undergoing a hip replacement procedure, willingness to have hip replacement surgery), logistic mixed-effects models will be fitted adjusted for the outcomes at baseline where able and BMI (> 27 kg/m^2^ and ≤ 27 kg/m^2^), with random effects for participant and physiotherapist, and results reported as risk ratios and risk differences at 6 months between the treatment groups. Analysis of moderation of the treatment effect by pre-specified potential moderators (baseline willingness to have surgery, BMI) on the two primary outcomes will be assessed by including interactions between moderators and the treatment group in the regression models. For all between-group comparisons, 95% confidence intervals and *p*-values will be reported. Standard diagnostic plots will be used to verify model assumptions.

### Patient and public involvement

End-users and stakeholders were engaged in developing the research question, study methodology, and intervention components. Representatives from our partner organisations, Medibank, Coviu, the Australian Physiotherapy Association and Dietitians Australia as well as a consumer with hip OA (JM) provided input into the research question and study protocol, and were also named Associate Investigators on the grant application. The Medibank marketing team conceived the name of the program. A consumer (JM) provided feedback about the length and ease of access of the initial proposed questionnaire battery, and was also named an Associate Investigator on the grant application. Four physiotherapists provided specific input into the design of the hip strengthening exercises while two consumers participated in the filming/production of the exercise videos. Coviu provided clinician and researcher training in the use of the online consultation platform and feedback on the physiotherapist and dietitian manuals and participant resources. The ‘My Exercise Messages’ app had extensive iterative engagement during the development of the behaviour change message library and app which has been previously described [39]. A dietitian from Dietitians Australia and an endocrinologist (PS) provided input into the design of the portion plate. Consumers and clinicians provided input and feedback on the participant resources.

### Timelines

The Human Research Ethics Committee of The University of Melbourne gave ethical approval on 23rd February 2022. We prospectively registered the trial with the Australian New Zealand Clinical Trials Registry on 24th March 2022. Participant recruitment commenced in June 2022. Recruitment is expected to be completed in June 2024. The main trial is due for completion in June 2025 when all participants have completed 12-month data.

### Dissemination

Study findings will be disseminated through conference presentations and publication in peer-reviewed journals as well as via our Centre website, knowledge translation network, media and social media including a study infographic. If the Better Hip program is found to be effective, Medibank will likely scale up the program as an offering across its broad membership base, by adapting implementation plans set in place with the previous scale-up of the *Better Knee, Better Me* program. Trial e-learning modules and clinician manuals will be made freely available on FutureLearn (a digital education platform) and via our Centre website for accessible clinician professional development. Downloadable patient resources will also be made freely available for clinicians and consumers. The International Committee of Medical Journal Editors recommendations for authorship will be followed.

## Discussion

This protocol describes the background, aims and methods for a two-group, parallel design RCT aiming to evaluate the effectiveness of a 6-month telehealth-delivered, clinician-supported lifestyle management program (*Better Hip*) on the primary outcomes of change in hip pain while walking and physical function at 6 months, compared with an information-only control for people with hip OA. The effects of the program on these primary outcomes at 12 months as well as on other clinical outcomes at 6 and 12 months will also be evaluated. A range of other measures will provide insights into the safety, feasibility, acceptability of and engagement with this innovative remotely-delivered model of service delivery that focuses on core recommended hip OA treatments of education, exercise/physical activity, and for those with overweight and obesity, weight loss. A separate health economic evaluation at 12-months will inform implementation decisions while a separate survey to the main trial will follow-up participants at 2-years to determine longer-term outcomes. Such a service model has the potential to increase patient access to evidence-based lifestyle management programs.

## Data Availability

The datasets used and/or analysed during the current study will be made available from the corresponding author on reasonable request.

## Appendix 1: Home-based strengthening exercise protocol*

Minimum of 5 and maximum of 7 exercises, with progression as appropriate:

- 1 x functional / quadriceps exercise
- 1 x hip abductor exercise
- 1 x hip flexor exercise
- 1 x hip extensor exercise
- 1 x hip external **OR** 1 x hip adductor exercise
- (+ if the participant is undertaking the dietary weight loss program: 2 x UL exercises)

Once a participant is able to do the minimum of 5 exercises, up to two additional exercises may be added from the above groups.

**Table:**

Muscle group	Exercise name	Starting position	Exercise instructions	Modification / progression options
Hip extensor exercises	Face-down-lying leg lift	Lie on your tummy with your hands folded under your chin.	Keep your hips flat on the floor/firm bed. Push your heel towards the ceiling, lifting your leg off the bed, and squeezing your buttocks the whole time. Hold for 2 seconds, and then lower your leg back to the starting position.	A pillow may be placed under the tummy for those who experience lower back discomfort.
	4-point-kneel leg lift	Kneel on your hands and knees. Place your hands directly under your shoulders and your knees directly under your hip. Lift your tailbone in the air a tiny, tiny bit so there is a very small curve in your back but your back is almost straight.	Keeping the tummy drawn in, slowly kick the study leg backwards, squeezing strongly through the buttocks. The knee should be kept at 90 degrees (i.e. short lever). Squeeze strongly through the buttock to get the leg in the air. Your thigh should go no further than parallel to your back. Bring leg back in, and repeat (no need to put the knee down on the ground between repetitions) **Keep your body very still**	Working with a cuff weight, resistance band or long lever.
	Bridge	Lie on your back with your knees bent and your feet flat on the floor/firm bed.	Squeeze your buttock muscles, tuck your tailbone under, and lift your hips and buttocks from the bed. Hold for 3 seconds. Slowly lower your bottom down to the floor/ bed. Keep your hips level during the exercise.	Perform the same exercise with the addition of a weight, as instructed by your physiotherapist.
	Split-leg bridge	Lie on your back with your knees bent and your feet flat on the floor/firm bed. Place your feet hip-width apart. Move your study leg slightly closer to your bottom and slightly in towards the center.	Keep your feet in the starting position. (Your study leg should be closer to your bottom and your non-study leg slightly further away). Lift your bottom. Take more weight through your study leg. Hold for 3 seconds. Then slowly lower your bottom down to floor/bed.	
	Double to single leg bridge	Lie on your back with your knees bent and your feet flat on the floor/firm bed.	1. Squeeze your buttock muscles, tuck your tailbone under and lift your hips and buttocks from the bed. 2. Keeping your hips level, lift your non-study leg off the floor/bed. Hold for 3 seconds. 3. Then place the non-study leg/foot back on the bed. 4. Slowly lower bottom back down to floor/bed	1. Progress duration of hold. OR 2. Move on to true single leg bridge as instructed below.
	Single leg bridge	Lie on your back with your knees bent and your feet flat on the floor/firm bed.	1. Lift your non-study leg off the floor/bed. Squeeze your buttock muscles and tuck your tailbone underneath you. 2. Keeping your hips level, lift your bottom and pelvis up off the bed using your study leg. 3. Hold for 3 seconds, then slowly lower bottom down to floor/bed. 4. Last of all, lower the non-study leg back to the floor/bed.	
	Hip raise	Lie on your back with your upper back supported on a step or firm couch, and your feet on the floor hip width apart.	Tighten your buttocks and push through your heels to bring your hips upward. Hold for 2 seconds, and then slowly lower your hips back to the starting position.	Progressions include adding a weight, or completing this exercise just on your study leg, as directed by your physiotherapist.
Functional / quadriceps exercises	Partial squats	Stand up tall with your legs shoulder- width apart. Turn your feet slightly outwards. Stay safe: Hold onto a table or chair for balance.	Bend at your hips and knees. Lower yourself down slightly, as if you were going to sit on a chair. Remember, when we sit down on a chair our bottom goes back behind us and down. Hold for 3 seconds. Slowly straighten back up.	
	Partial squats against wall	Gently lean your back against a wall. Keep your buttocks, back, and shoulders resting against the wall during the exercise. Step your feet away from the wall (about 30 cm) with your feet hip-width apart. Turn your feet slightly outwards and try to keep the weight on your heels.	Slowly slide down the wall. Keep your heels on the ground. Keep your knees in line with your feet, trying not to let your knees collapse inwards Stop before your knees go past your toes (or less if it is painful). Hold for 3 seconds. Push through your heels and slowly slide back up the wall.	1. Addition of resistance band around your knees/ Push your knees out against the resistance band. Try not to let them collapse in during the exercise. 2. Half-way holds: hold for 3 seconds at the halfway point of the exercise while going up and/or down.
	Split leg wall squats	Gently lean your back against a wall. Keep your buttocks, back, and shoulders resting against the wall at all times during the exercise. Step your feet away from the wall (about 30 cm) with your feet hip-width apart and your weight in the heels. Move your non-study leg a further 15 cm away from the wall. Your study leg will be slightly behind your non-study leg, and this will place more load/weight through your study leg.	Slowly slide down the wall. Take more weight through your study leg (the leg closest to the wall). Keep your heels on the ground. Keep your knees in line with your feet, trying not to let your knees collapse inwards. Stop before your knees go past your toes (or sooner if it is painful). Hold for 3 seconds, and push through your heels and slowly slide back up the wall.	
	Sit to stand	Sit on a firm, stable chair. Place the chair back against a wall for support if needed. Place your feet shoulder width apart.	Slowly stand with your hands crossed over your shoulders, or resting on your lap. Start by leaning forward bringing your nose over your toes. Keep your knees in line with your toes. As you lift up from the chair, push through the heels and straighten your legs until you are standing completely straight. Sit back down slowly.	1. Addition of resistance band around your knees (as shown at right) Push your knees out against the resistance band, trying not to let them collapse in during the exercise. 2. Half-way holds: Hold for 3 seconds at the halfway point of the exercise while going up and/or down. 3. Use a lower chair 4. Hold a weight
	Sit to stand with more weight on study leg	Sit in a firm, stable chair. Place the chair back against a wall for support if needed. Place your feet shoulder width apart. Take more weight on your study leg by either: (a) placing your good leg further forward so that your study leg is closer to you, or (b) shifting both your feet sideways so your study leg is lined up with the middle of your body.	Push through the heel of your study leg and slowly stand up from the chair without using your hands. Keep your knee in line with your foot during the exercise. Try to have more than half of your body weight on your study leg throughout the entire exercise Slowly return to sitting.	
	Step ups	Stand in front of a stair or step. Stay safe: Use a handrail or other hand support for balance if required.	Place your study leg up onto the step. When you are stable, push through your heel/ foot and bring up your other leg. Lightly touch your non-study leg onto the step, and step it back down slowly to the start position. Your weight should be on your study leg throughout the entire exercise. Concentrate on keeping your knee positioned over your foot throughout.	1. Use a higher step. 2. Hold on to a weight.
	Backward step downs	Stand on a step with your study leg close to the edge of the step. Stay safe: Use a handrail or other hand support for balance if required.	Bending your study leg slowly, sit your buttocks back and lean forward slightly. Make sure the knee on the study leg side is not bending/drifting in towards the midline. Lightly touch the foot of the non-study leg to the ground and then push back up to the starting position.	1. Use a higher step. 2. Hold on to a weight. 3. Use a cuff weight on the non-study leg
	Lunges	Stand with your feet shoulder-width apart and your arms either by your side or resting on your hips.	Take a large step forward with your study leg, then lower your hips down, bending your hips and knees to about 90 degrees. Keep your upper body upright, and don’t let the knee that’s forward drift inwards towards the midline. Push back up and step back to return to the starting position.	Hold weights at your sides.
	Inner-range quads over roll	Lie on a mat on the floor or on a firm bed. Put a rolled up towel under your study leg knee. Your knee will be slightly bent. Keep the knee cap and toes pointing toward the roof.	Keeping the back of the knee in contact with the towel, push the back of your knee down into the towel and straighten your study leg and SLOWLY lift the heel off the surface over 2 seconds. Hold leg as straight as it will go for 5 seconds then SLOWLY lower down over 2 seconds.	
	Seated knee extension	Sit in a firm chair (one that is higher if possible).	Slowly lift your foot up and straighten the knee until it is fully straight. Keep the back of your thigh on the chair. Hold for 5 seconds and lower slowly. *“Slowly up, hold, 2, 3, 4, 5, slowly down”.*	Tie your resistance band into a loop. Place the looped resistance band around the back leg of a chair. Sit on the chair and put your leg into the loop with the band around the front of your foot. Change colour of resistance band – red through to black. Progressions also include adding an ankle cuff weight (as guided by your physiotherapist).
Hip abductor exercises	Standing side leg raises	Face forwards and keep your back straight. Loop your resistance band around your ankles. Stay safe: Use the back of a chair or a wall for balance.	Stand tall, keeping your back straight. Try not to tilt to the side. Don’t twist as this will mean the wrong muscles are being exercised. Keep your knees straight and your toes pointing forward. Squeeze through your buttock and leg and lift your study leg out a small way to the side, leading with the heel. Hold for 1–3 seconds and then lower slowly.	Alternate option: Use an ankle cuff weight instead of resistance band. Easier option (as guided by your physiotherapist): you may do this same exercise without the use of the resistance band.
	Standing leg wall press	Stand sideways with your non-study leg against a sturdy wall.	Stand tall, tummy and bottom in. Lift the non-study leg off the floor so that your hip, thigh and knee are touching the wall. Keeping your body still, push your non-study leg into the wall. Hold for 3–5 seconds, or as directed by your physiotherapist. Return your foot to the floor and rest for a few seconds.	
	Standing leg wall press with knee bend	Stand sideways with your non-study leg against a sturdy wall.	Stand tall, tummy and bottom in. Lift the non-study leg off the floor so that your hip, thigh and knee are touching the wall. Keeping your body still, push your non- study leg into the wall. While continuing to push into the wall, slowly bend your study leg to a maximum of 45 degrees. Concentrate on keeping your knee positioned over your foot throughout. Straighten your knee and return your foot to the floor and rest for a few seconds.	
	Side-lying leg raises	Lie on your side on a firm surface, with your study leg on top. You may bend the bottom leg and use your arms for support. The body and hips should be rolled forwards, about a 1/4 turn forwards. This step is important; you should feel that you are rolling forwards slightly. This position of the body and hips should be maintained throughout the entire exercise.	Think about a strong still body and drawing your leg into the hip socket. Slowly raise the top (study) leg up, keeping your knee facing forwards. Make sure your body does not roll backwards during the exercise. Hold for 1–3 seconds, then lower down slowly.	Advanced option (As guided by your physiotherapist) Addition of the cuff weight to the leg.
	Crab walking	Place a resistance band around your thigh/knee level (easier) or around your ankles (harder) so that there is tension when your legs are separated about 10 cm. Slightly bend both knees. Stay safe: You should stand facing a table, a kitchen bench or a wall that you can reach if you lose balance.	Step sideways against the tension of the resistance band, keeping your legs apart. Do not twist or turn your body and legs. Your feet should point forwards while you are stepping sideways. Concentrate on keeping your knee positioned over your foot throughout the exercise. Take 3 steps to the left, and then 3 steps to the right. Continue for 30 seconds to complete 1 round.	Advanced options: 1. Zig-zags 2. Change resistance band colour
	Hip hitch	Stand side-on on a step, with your study leg on the step and the non-study leg hanging off the step. Stay safe: hold onto a wall or handrail.	Bend the knee on the study leg very slightly. Lower the leg that is hanging off the step by lowering that side of the pelvis down towards the floor. Push back up through the study hip to the starting position.	Using a cuff weight on the non-study leg.
	Bridge with outward leg press	Lie on your back with your knees bent and your feet flat on the floor / firm bed. Put a resistance band around your legs just above your knees as shown.	Squeeze your buttock muscles, tuck your tailbone under and lift your hips and buttocks from the floor / bed. Push your legs out against the resistance of the resistance band, holding for 1 second. Then bring your legs slowly back together and lower your buttocks down to the floor / bed.	Advanced options: 1. Change to a harder resistance band colour. 2. Push out against the resistance band more than once while the buttocks are lifted.
Hip flexor exercises	Crook lying hip bends	Lay on a firm surface/bed, with both knees bent and feet resting flat on the bed.	Think about drawing your leg up into the hip joint and slowly raise your study leg into the air. Slowly lower your study leg down. This exercise should be slow and controlled. You are aiming to work the muscles deep in the front of the hip.	Advanced Options: 1. Addition of cuff weight to the study leg, just above the knee. 2. Straighten the knee (long lever lifts)
	Face up lying hip bends off edge of bed	Lay on a firm surface/bed, with your study leg hanging off the end of the bed. Bring the knee of your non-study leg to your chest, and secure with your arms Stay safe: It is very important you make sure you tuck your tail under and keep your back flat against the bed. You should only feel this exercise in your hip/front of your thigh, not in your back at all. It may also work your stomach muscles a little.	Tuck your tail under, making sure your back is flat against the bed. Think about sucking in the leg into the hip joint, and slowly raise your study leg into the air. Keep the knee bent on your study leg. Slowly lower your study leg down towards the level of the bed but no further.	Advanced options: 1. Add a cuff weight to the study leg, just above the knee. Straightening the knee of the study leg (long lever).
	Standing knee raises	Stand tall with your legs shoulder width apart. Stay safe: Use a chair or table for balance.	Standing tall and strong, bend your study leg up so that your thigh is parallel to the ground (or lower if discomfort is experienced). Slowly lower your leg back down. Lightly touch your foot to the ground before repeating straight away. The aim of this exercise is to progressively build speed, while keeping all the movement at the hip joint. Your body (trunk and pelvis) should not be rocking backwards or forwards during the exercise.	Progressions include adding an ankle cuff weight or resistance band.
Hip adductor exercises	Crook lying leg squeeze	Lay on a firm surface with your knees bent. Keep you tour tail tucked under so your back is comfortably flat against the bed/floor. Keep your heels on the floor/bed. Place a magic circle, ball, cushion or other appropriate equipment between the knees.	You are aiming to start to work the muscles on the inside of your thighs. Gently squeeze your knees together and build up to a moderate pressure. Hold for 5 seconds, or as directed by your physiotherapist.	
	Standing resistance band adduction	Tie the resistance band to a stable support and loop it around your ankle as shown. Step away from the support to create some tension in the resistance band. The non-study leg will stay still during the exercise. Stand up straight. Keep your body facing forwards (at right angles to the band) throughout the exercise. Stay safe: use a chair or table for balance as required.	Standing tall and keeping your belly drawn in, slowly move your study leg to your midline pulling against the resistance band. Keep all your body weight supported on your non-study leg. Slowly return your study leg to the starting position.	
	Side lying hip adduction	Lie on your side on your study hip with a small rolled up towel between your rib cage and waist. Place your non-study foot in front of your knee so it is out of the way of / not on top of your study leg. (If too difficult, just lie with legs one on top of the other). Your study leg will be straight, toes pulled up towards you, knee facing forwards.	Keeping your body very still, think about sucking your thigh/leg up into the hip joint socket and slowly lift the study leg up off the bed. Keep your knee straight. This is a very small movement and there is no need to overdo it - you are aiming to simply get your foot off the ground. You should feel the work in your inner thigh of the study leg. Slow controlled lift up, slow controlled lower down.	Progressions include adding an ankle cuff weight.
	Crook lying resistance band adduction	Lie on your back with your knees bent and your feet flat on the floor / firm bed. Put a resistance band around your study leg just above the knee and attach it to a secure object like a table leg or bed. Start with your study leg falling slightly out towards the pull of the band.	Squeezing the inside of the thigh on your study leg, bring the study leg into the midline against the pull of the resistance band. Hold for 3 seconds and then slowly return to the starting position.	Advanced options: 1. Change to a harder resistance band. 2. Add a bridge while holding your leg in the midline: squeeze your buttock muscles, tuck your tailbone under and lift your hips and buttocks from the floor. Then lower your buttocks down and return to the starting position.
Hip external rotator exercises	Lying leg rotations	Lie on your front on a firm surface with a resistance band around the ankle of your study leg. Attach the band to a secure object like a table leg or bed.	Bend the knee on your study leg to 90 degrees and allow the lower leg to fall out with the pull of the resistance band. Keep your thighs in the same position while you rotate the lower leg inward to the midline, pulling against the band. Hold for 3 seconds in this position. Control the leg as you rotate it back to the starting position.	Change to a harder resistance band as guided by your physiotherapist.
	Hip aeroplanes	Start standing in front of a bench, couch or bed, with your feet hip width apart.	Standing only on the study leg, left the other leg up behind you as you tip forward. Try and make your lifted leg and torso in a straight line horizontal to the ground. Only use your hands on the bench/couch/bed as you need to for support. Rotate your pelvis and upper body slowly away from the study leg by pushing through your study leg, and then slowly lower the pelvis to the starting position. Stand back upright, bringing both feet back to the ground.	Advanced options: 1. Complete the exercise without holding onto the bench/couch/bed for support. 2. Add a cuff weight to the non-study leg.
Balance exercises	Tandem stance	Stand on a firm surface. Look forwards focusing on a point on the wall. Use hand support (e.g. a chair) for balance if required.	Place 1 foot in front of the other so that feet make a straight line. Hold for 10 seconds. *“Hold, 2, 3, 4, 5, 6, 7, 8, 9, relax”.* Switch foot position so that the foot that was in front is now in the back. Hold for 10 seconds. *“Hold, 2, 3, 4, 5, 6, 7, 8, 9, relax”.*	1. Let go of hand support (if required) as you feel stable. 2. Maintain balance while slowly raising arms in the air. 3. Eyes closed.
	Forwards tap	Stand on a firm surface. Use hand support (e.g. a chair) for balance if required.	Slowly tap forwards and back with the ‘tapping leg’ (non-arthritis leg) while balancing on the arthritis leg. Start with tapping just a few inches forwards and backwards and progress to larger taps as you gain control. Keep your weight on the arthritis leg. Concentrate on keeping your knee positioned over your foot throughout.	
	Single leg balance	Stand with your feet shoulder width apart. Stand close to a wall for support in case you over balance, if required. Use hand support (e.g. a chair) for balance, if required.	Stand on single leg. Try to hold for 10 seconds. *“Lift, 2, 3, 4, 5, 6, 7, 8, 9, relax”.* Keep your weight on the arthritis leg. Concentrate on keeping your knee positioned over your foot throughout.	1. Increase hold time for up to 30 secs, as you feel stable. 2. Maintain balance while slowly raising and lowering your arms in the air. 3. Eyes closed.
Arm strengthening exercises (prescribed for those undergoing weight loss intervention)	Bicep curls	Stand with your back straight and your arms by your sides. Hold a small weight or half a litre of water in each hand (start with a weight of around 500 g)	Slowly bend your right elbow up to bring your hand toward your shoulder, then slowly lower back down to the starting position. Repeat on the left side. Perform one set of 10–15 repetitions for each side. If you are comfortable with this exercise you may be able to increase to 2 sets, 3 times per week and then add more weight - up to around 2 kg.	
	Wall push ups	Stand facing a wall, with your feet shoulder- width apart and about 30cms away from the wall. Place your palms on the wall around shoulder height.	Slowly bend your elbows so that you lean in towards the wall. Slowly straighten your elbows to push yourself back to the starting position. Perform one set of 10–15 repetitions, 3 times per week. If you are comfortable with this exercise you may be able to increase to 2–3 sets of 15 repetitions, 3 times per week.	Progression: Complete exercise from a kitchen bench/table

* Adapted from: Hall M, Hinman RS, Knox G, et al. Effects of adding a diet intervention to exercise on hip osteoarthritis pain: protocol for the ECHO randomized controlled trial. *BMC Musculoskeletal Disorders.* 2022/03/052022;23 [1]:215

## Abbreviations

ANZCTR: Australian New Zealand Clinical Trials Registry
AQoL-8D: Assessment of Quality of Life instrument – 8 dimension
BEACH: Bettering the Evaluation and Care of Health
BMI: Body Mass Index
CHESM: Centre for Health, Exercise and Sports Medicine
CONSORT: Consolidated Standards of Reporting Trials
CI: Confidence Interval
GP: General Practitioner
HOOS: Hip dysfunction and Osteoarthritis Outcome Score
HREC: Human Research Ethics Committee
MCID: Minimal Clinically Important Difference
NHMRC: National Health and Medical Research Council
NIH: National Institutes of Health
NRS: Numerical Rating Scale
OA: Osteoarthritis
OARSI: Osteoarthritis Research Society International
PASE: Physical Activity Scale for the Elderly
PLS: Plain Language Statement
QALY: Quality-Adjusted Life Years
RCT: Randomised Controlled Trial
SPIRIT: Standard Protocol Items: Recommendations for Interventional Trials
VLED: Very Low Energy Diet
WOMAC: Western Ontario and McMaster Universities Osteoarthritis Index
